# Increased Serum Level of MicroRNA-663 Is Correlated with Poor Prognosis of Patients with Nasopharyngeal Carcinoma

**DOI:** 10.1155/2016/7648215

**Published:** 2016-09-07

**Authors:** Shaoqiang Liang, Ning Zhang, Yanming Deng, Lusi Chen, Yang Zhang, Zhenhe Zheng, Weijun Luo, Zhiqian Lv, Shaoen Li, Tao Xun

**Affiliations:** Department of Radiotherapy, Tumor Hospital of First People's Hospital of Foshan, Foshan, Guangdong 528041, China

## Abstract

MicroRNAs (miRs) play crucial roles in the carcinogenesis and malignant progression of human cancers including nasopharyngeal carcinoma (NPC). In this study, we aimed to investigate the association of serum miR-663 levels with the clinical factors and prognosis of NPC patients. Real-time PCR was performed to examine the amount of miR-663 in serum in NPC patients and healthy controls. Our data showed that the amount of miR-663 in serum was significantly higher in NPC patients than in healthy controls. Moreover, the serum levels of miR-663 were significantly correlated with the grade, lymph node metastasis, and clinical stage of NPC. Furthermore, higher serum miR-663 levels were closely associated with worse 5-year overall survival (OS) and relapse-free survival (RFS) of patients with NPC, and the serum level of miR-663 was found to be an independent predicator for the prognosis of NPC. In addition, after receiving chemoradiotherapy, the serum levels of miR-663 were significantly reduced in NPC patients. In summary, miR-663 was upregulated in the serum of NPC patients, which was downregulated after chemoradiotherapy, and its increased levels were closely associated with malignant progression and poor prognosis in NPC patients. Therefore, the amount of miR-663 in serum may become a potential predicator for the clinical outcome of NPC patients.

## 1. Introduction

Nasopharyngeal carcinoma (NPC), a head and neck cancer, is most common in Southeast Asia especially in Southern China, and the incidence rate of NPC is approximately 20 cases per 100,000 people in endemic areas [1, 2]. The pathogenesis of NPC involves Epstein-Barr virus (EBV) infection and genetic susceptibility, and chemoradiotherapy is the standard approach for the treatment of NPC [3, 4]. Moreover, the detection of NPC mainly relies on tissue biopsy and cancer imaging, but its early diagnosis is difficult due to its anatomical location [5, 6]. Therefore, it is urgently needed to explore effective biomarkers for early detection of NPC, as well as predication of the prognosis of NPC patients.

MicroRNAs (miRs) are a kind of small noncoding RNAs, containing 22–25 nucleotides [7]. They generally play suppressive role in regulating the gene expression through directly binding to the 3′ untranslational region (UTR) of their target mRNAs, causing translation inhibition or mRNA degradation [7]. In recent years, miRs have been implicated in various cellular biological processes, including cell proliferation, apoptosis, differentiation, metabolism, motility, and tumorigenesis [7–9]. They are involved in the regulation of the expression of many oncogenes or tumors suppressors and thus play promoting or suppressive roles in human cancers [10, 11]. In addition, deregulations of miRs have been found in NPC [12, 13]. Wang et al. reported that miR-429 was downregulated in NPC and played a suppressive role in NPC cell migration and invasion [14]. Mao et al. found that miR-205 could promote the proliferation, migration, and invasion of NPC cells through activation of AKT signaling [15]. MiR-24 was demonstrated to enhance the radiosensitivity of NPC cells by targeting SP1 [16].

MiR-663 has previously been reported to be frequently deregulated in several types of human cancers, indicating that miR-663 may play an important role in carcinogenesis. The expression levels of miR-663 were significantly decreased in gastric cancer [17]. However, it was found to be markedly upregulated in castration-resistant prostate cancer [18]. Recently, it was reported that miR-663 was upregulated in NPC tissues and cell lines, and knockdown of miR-663 suppressed the growth of NPC cells in vitro and in vivo [19]. However, evidence in the serum levels of miR-663 in NPC as well as its clinical significance still remains limited.

In this study, we aimed to investigate the clinical significance of serum miR-663 levels in NPC, as well as its predictive value for prognosis of NPC patients.

## 2. Materials and Methods

### 2.1. Ethics Approval

The present study was approved by the Ethics Committee of First People's Hospital of Foshan, Foshan, China. All the participants involved in this study have written the written consent.

### 2.2. Study Population

A total of 74 cases of serum samples from NPC patients and 27 healthy controls were recruited at the Department of Radiotherapy, Tumor Hospital of First People's Hospital of Foshan. The ages of the NPC patients including 40 male and 34 female were ranged from 28 years to 71 years. The clinical characteristics of NPC patients were summarized in Table 1. Before sample collection, all the participants did not receive any therapy. The serum was isolated from 10 mL of blood by centrifuging at 1,000 ×g at room temperature for 5 min and then at 12,000 ×g at 4°C for 5 min. The samples were stored at −80°C before usage.

### 2.3. Real-Time Polymerase Chain Reaction

QIAamp RNA Blood kit (Qiagen, Hilden, Germany) was used to extract the total RNA, according to the manufacturer's instruction. miRNA Reverse Transcription Kit (Life Technologies) was used to convert 1 *μ*g of RNA into cDNA, according to the manufacturer's instruction. Real-time PCR was then performed by using a miRNA Q-PCR Detection Kit (GeneCopoeia, Rockville, MD, USA) on ABI 7500 thermocycler (Applied Biosystems, Carlsbad, CA, USA). The PCR conditions were 95°C for 5 min, followed by 40 cycles of denaturation at 95°C for 15 s, 58°C for 30 s, and 72°C for 30 s. Each sample was examined in triplicate and U6 gene was used as an internal reference. The relative expression of miR-663 was analyzed by the 2^−ΔΔCt^ method that normalized to U6 expression.

### 2.4. Statistical Analysis

Data were expressed as means ± standard deviation (SD). SPSS 20.0 software (SPSS Inc., IL, USA) was used to perform the statistical analysis. Nonparametric *t*-test was used to analyze the differential expression of serum miR-663 between NPC patients and healthy controls. Chi-square test was used to analyze the association between the serum level of miR-663 and clinicopathological parameters of NPC. Kaplan-Meier analysis with the log-rank test was used to examine the association between the serum level of miR-663 and the 5-year overall survival (OS) and relapse-free survival (RFS). Cox proportional hazard regression model was used to estimate the independent predicators for the prognosis of NPC patients. Paired sample *t*-test was used to analyze the differential amount of miR-663 in serum between NPC patients before treatment and those after treatment. *P* value less than 0.05 was considered statistically significant.

## 3. Results

### 3.1. The Serum miR-663 Levels Are Higher in NPC Patients Than in Healthy Control

**Figure 1:**
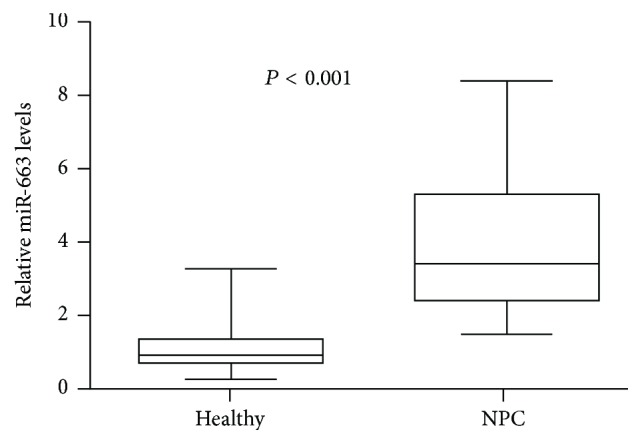
Real-time PCR was used to detect the serum levels of miR-663 in 74 nasopharyngeal carcinoma (NPC) patients and 27 healthy controls. Box-whisker plot (bottom to top) means the minimum, lower quartile, median, upper quartile, and maximum value.

In our study, we firstly examined the levels of miR-663 in serum from NPC and healthy controls using real-time PCR. Our data indicated that the serum miR-663 levels were significantly higher (about 3.5-fold) in NPC patients than in healthy control (*P* < 0.001, Figure 1).

### 3.2. The Serum miR-663 Levels Are Significantly Associated with Grade, Clinical Stage, and Lymph Node Metastasis in NPC

We further studied the association between the serum miR-663 levels and clinical characteristics in NPC patients. All NPC patients were divided into two groups, high serum levels of miR-663 group and low serum levels of miR-663 group, according to the mean expression of serum miR-663 as a cut-off point. As demonstrated in Table 1, the serum miR-663 levels were not correlated with age (*P* = 0.980), gender (*P* = 0.939), T stage (*P* = 0.112), distant metastasis (*P* = 0.172), and EBV infection (*P* = 0.239). However, it was significantly associated with the grade (*P* = 0.013), clinical stage (*P* = 0.001), and lymph node metastasis (0.001). Thus, our data suggest that the serum levels of miR-663 may be used as a biomarker for evaluating the malignant progression of NPC.

### 3.3. Higher Serum Level of miR-663 Is Associated with Poor 5-Year Survival Rates in NPC Patients

**Figure 2:**
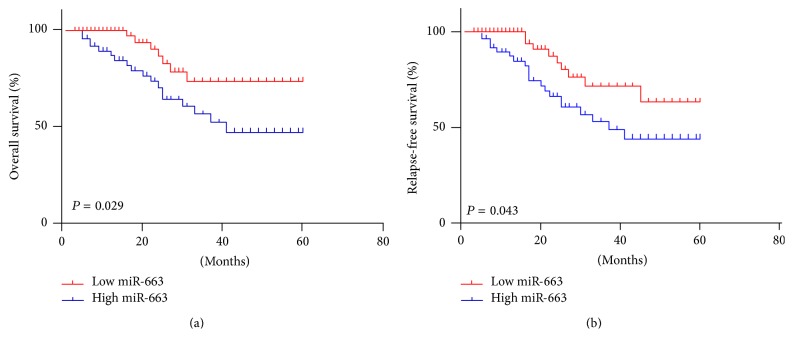
The relationship between serum level of miR-663 and 5-year overall and relapse-free survival rates of nasopharyngeal carcinoma patients.

 We further investigated the association between the serum level of miR-663 and the survival rate in NPC patients using the Kaplan-Meier method. We found that the NPC patients with higher serum level of miR-663 showed shorter 5-year overall survival (Figure 2(a), OS, *P* = 0.029) and relapse-free survival (Figure 2(b), RFS, *P* = 0.043), when compared to those with lower serum level of miR-663. Therefore, higher serum level of miR-663 is associated with poor prognosis of NPC patients.

### 3.4. The Serum Level of miR-663 Is an Independent Predicator for the Prognosis of NPC Patients

We further investigated the factors that could predicate the prognosis of NPC patients by using the univariate and multivariate analyses. Univariate analysis data indicated that the serum level of miR-663 (*P* = 0.014), as well as the grade (*P* = 0.021), lymph node metastasis (*P* = 0.009), and clinical stage (*P* = 0.001), was significantly associated with the survival (Table 2). Moreover, as demonstrated in Table 3, the serum level of miR-663 (*P* = 0.025), lymph node metastasis (*P* = 0.035), and clinical stage (*P* = 0.001) were found to be independent factors for predicating the prognosis of NPC patients.

### 3.5. The Association between Serum Level of miR-663 and Treatment Response

**Figure 3:**
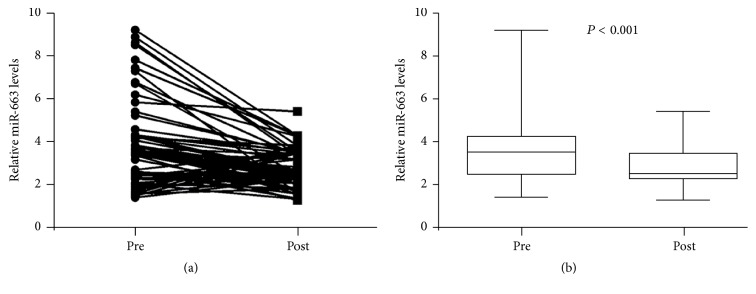
(a and b) Real-time PCR was used to detect the serum level of miR-663 in 74 nasopharyngeal carcinoma patients before and after receiving chemoradiotherapy. Box-whisker plot (bottom to top) means the minimum, lower quartile, median, upper quartile, and maximum value. Pre, pretreatment; post, posttreatment.

Finally, we compared the serum levels of miR-663 between the NPC patients before treatment and after treatment. As indicated in Figures 3(a) and 3(b), we found that the serum levels of miR-663 were remarkably decreased in NPC patients after receiving the standard chemoradiotherapy (*P* < 0.001). These data suggest that the serum levels of miR-663 may be used as an important biomarker for monitoring the treatment response.

## 4. Discussion

The prognosis of patients with stages III and IV NPC was worse when compared with that of patients with stages I and II NPC [20]. Therefore, it is urgently needed to identify novel biomarkers for the early detection of NPC. Recently, the novel biomarkers in the body fluids, such as plasma, serum, saliva, and urine, show promises for the early detection of malignancies including NPC. miRs have been demonstrated to play crucial roles in the development and malignant progression of human cancers. Moreover, accumulating evidences have shown that some serum miRs may become important biomarkers not only for the early detection of human cancers, but also for the evaluation of cancer progression or treatment responses. For instance, the serum expression of miR-199a was significantly decreased in patients with epithelial ovarian cancer, which was significantly associated with the tumor stage, lymph node metastasis, and distal metastasis, suggesting that the downregulation of miR-199a may be a potential indicator for disease progression [21]. Besides, the circulating miR-92a, miR-100, and miR-143 were recently identified as noninvasive biomarkers for bladder cancer diagnosis [22].

MiR-663 has recently been found to generally play a suppressive role in several kinds of human cancers. The expression of miR-663 was significantly lower in pediatric acute myeloid leukemia (AML) cells compared to normal bone marrow due to the hypermethylated promoter, suggesting that downregulation of miR-663 may be involved in the development of AML [23]. Li et al. reported that miR-663 was downregulated in glioblastoma tissues and had inhibitory effects on the proliferation, migration, and invasion of glioblastoma cells via targeting TGF-*β*1 [24]. Similar findings were also reported that miR-663 acted as a tumor suppressor in glioblastoma by targeting CXCR4 and PIK3CD [25, 26]. Zang et al. indicated that miR-663 could inhibit the tumor growth and invasiveness in pancreatic cancer by directly targeting EEF1A2 [27]. Moreover, treatment with waltonitone in non-small cell lung cancer led to an upregulation of miR-663, which further caused downregulation of bcl-2 and induces cancer cell apoptosis [28]. Overexpression of miR-663 significantly decreased the proliferation, migration, and colony formation of multiple myeloma cells [29]. Besides, other cancer-related factors were also found to be direct targets of miR-663, such as EEF1A2 and HSPG2 [30, 31].

On the contrary, however, several studies also reported that miR-663 played a promoting role in several cancer types [32]. Sand et al. reported that miR-663 was significantly upregulated in cutaneous malignant melanoma compared to benign melanocytic nevi [32]. Liu et al. showed that miR-663 was highly expressed in lung cancer patients and promoted the proliferation of lung cancer cells by directly or indirectly regulating TGFB1, P53, Bax, and Fas [33]. Therefore, it seems that the role of miR-663 is cancer-specific, and miR-663 plays dual roles in different cancer types, probably because it regulates different target genes in different tumor microenvironments. In the present study, we found that the serum miR-663 levels were significantly higher in NPC in patients than in healthy control. Moreover, its expression levels were significantly associated with several important clinicopathological features including grade, lymph node metastasis, and clinical stage, suggesting that the upregulation of serum level of miR-663 may be used as an important biomarker for the malignant progression of NPC. Besides, we further found that NPC patients with higher serum miR-663 levels showed poorer 5-year OS and RFS when compared with those with lower serum miR-663 levels and that serum miR-663 was an independent predicator for the prognosis of NPC. These findings highlight the importance of serum miR-663 in the clinical application in evaluating the prognosis of NPC patients. Consistent with our study, the expression of miR-663 was also found to be increased in NPC tissues and cell lines. Molecular mechanism investigation revealed that miR-663 could promote the proliferation of NPC cells in vitro and in vivo by directly targeted p21(WAF1/CIP1) to promote the cellular G1/S transition [19].

Finally, we found that the serum levels of miR-663 were remarkably decreased in NPC patients after receiving the standard chemoradiotherapy. Recently, miR-774 was also reported to be upregulated in the serum of NPC patients, and its expression level was significantly reduced after the patients had received the standard chemoradiotherapy [34]. Therefore, the serum levels of both miR-663 and miR-774 may be used as important biomarkers for monitoring the treatment response.

## 5. Conclusion

Our study for the first time demonstrates that the serum expression miR-663 is upregulated in NPC, and high serum miR-663 levels are significantly associated with the malignant progression and poor prognosis in NPC patients. Therefore, the serum level of miR-663 may become a potential predicator for the clinical outcome of NPC patients.

## Figures and Tables

**Table 1:** Association between serum miR-663 levels and clinicopathological characteristics of patients with nasopharyngeal carcinoma.

Factors	Number	Low miR-663 (*n* = 41)	High miR-663 (*n* = 33)	*P* value
Age				
<55	36	20	16	0.980
≥55	38	21	17	
Gender				
Male	40	22	18	0.939
Female	34	19	15	
Grade				
G1-2	41	28	13	0.013
G3	33	13	20	
T stage				
T1-2	39	25	14	0.112
T3-4	35	16	19	
Lymph node metastasis				
No	41	30	11	0.001
Yes	33	11	22	
Distant metastasis				
No	53	32	21	0.172
Yes	21	9	12	
Clinical stage				
I-II	39	29	10	0.001
III-IV	35	12	23	
EBV infection				
No	8	6	2	0.239
Yes	66	35	31	

**Table 2:** Univariate analysis of prognostic factors of NPC.

Factors	Hazard ratio	*P* value
Age (≥55/<55)	1.12	0.867
Gender (male/female)	1.1	0.882
Grade (G3/G1-2)	2.73	0.021
T stage (T3-4/T1-2)	1.63	0.115
Lymph node metastasis (yes/no)	3.45	0.009
Distant metastasis (yes/no)	1.61	0.124
Clinical stage (III-IV/I-II)	5.42	0.001
EBV infection (yes/no)	1.48	0.343
Serum miR-663 levels (high/low)	3.88	0.014

**Table 3:** Multivariate analysis of independent prognostic factors of NPC.

Factors	Hazard ratio	*P* value
Grade	1.73	0.077
Lymph node metastasis	2.43	0.035
Clinical stage	4.32	0.001
Serum miR-663 levels	2.68	0.025
